# OLIG2 expression level could be used as an independent prognostic factor for patients with cerebellar Glioblastoma (cGBM)

**DOI:** 10.1016/j.clinsp.2022.100120

**Published:** 2023-03-30

**Authors:** Jia Zhou, Ling-Fei Shi, Zheng Wang, Min Li, Jin-Seng Zhang, Ying Mao, Wei Hua

**Affiliations:** a Department of Neurosurgery, Huashan Hospital, Fudan University, Shanghai, China; b Cancer Center, Department of Neurosurgery, Zhejiang Provincial People's Hospital (Affiliated People's Hospital, Hangzhou Medical College), Hangzhou, Zhejiang, China; c Department of Geriatics, Zhejiang Provincial People's Hospital (Affiliated People's Hospital, Hangzhou Medical College), Hangzhou, Zhejiang, China

**Keywords:** Cerebellar Glioblastoma Multiforme, OLIG2, Biomarkers, Molecular pathology

## Abstract

**Objectives:**

The incidence of cerebellar Glioblastoma Multiforme (cGBM) is rare. Database like TCGA have not distinguish cGBM from GBM, our knowledge on cGBM gene expression characteristics is limited. The expression status of Oligodendrocyte Lineage Transcription factor 2 (OLIG2) and its clinical significance in cGBM is still unclear.

**Methods:**

The clinical data and tissue specimens of 73 cGBM patients were retrospectively studied. The association between OLIG2 expression level and the demographic characteristics of cGBM patients was identified by the Chi-Square test. The survival curves were drawn by Kaplan-Meier analysis. The independent prognostic factors was calculated according to Cox regression analysis.

**Results:**

The OLIG2 high expression was observed in about 57.5% (42/73) of the cGBM patients. Patients with high OLIG2 expression levels had a higher alive ratio at the end of follow-up (alive ratio: 70.6% vs. 29.4%, *p* = 0.04). The median survival time was 21 months and 13 months for high and low expression of OLIG2 (*p* < 0 .05). Univariate analysis and Multivariate analysis indicated that EOR (HR = 3.89, 95% CI 1.23‒12.26, *p* = 0.02), low OLIG2 expression (HR = 5.26, 95% CI 1.13‒24.59, *p* = 0.04), and without adjuvant therapy (HR = 4.95, 95% CI 1.22‒20.00, *p* = 0.03) were independent risk factors for the OS of cGBM patients.

**Conclusion:**

High expression level of OLIG2 could be used as an independent favorable prognosis indicator in cGBM patients and be recognized as a characteristic biomarker of cGBM.

## Highlights

• The retrospective study included 73 cGBM patients, which was the largest patient cohort in east Asia to date.
• OLIG2 expression level was high in more than half cGBM patients (42/73, 57.5%).
• OLIG2 expression level could be used as an independent prognostic factor for cGBM patients.

## Introduction

The incidence of Glioblastoma Multiforme (GBM) ranks first in human primary malignant brain tumors.^1^ However, cerebellar Glioblastoma Multiforme (cGBM) is quite rare, its incidence is around 0.4‒3.4% among all GBM.^2^^,^^3^ Based on previous studies, researchers found some unique clinical characteristics of cGBM and pointed out it was a special subtype of GBM.^4^, ^5^, ^6^, ^7^ In the last decades, researchers also found unique molecular profiling of GBM,^8^, ^9^, ^10^ GBM is highly heterogeneous on gene expression profiles and can be classified into 4 distinct molecular subtypes: proneural (Oligodendrocyte Progenitor [OPC] signature), classical (astrocytic signature), neural (neuronal signature), and mesenchymal (reactive astrocyte and microglia signature),^11^ these tumor molecular characteristics had importance for GBM patient's prognosis and precise treatment.. Unfortunately, tumor databases such as TCGA did not distinguish cGBM from supratentorial Glioblastoma Multiforme (sGBM). So it's important to explore unique molecular biomarkers of cGBM that correlate with clinical significance and to promote the search for a potential therapeutic target and strategy against this lethal disease.

OLIG2 (basic helix-loop-helix transcription factor) is mainly expressed in cell nuclei and is a Central Nervous System (CNS) restricted transcription factor that plays a critical role in glial progenitor proliferation.^12^ But the role of OLIG2 in cGBM remains uncertain.

The presented study included 73 cGBM patients, the largest case series in Asia to date. The aim of the study was to find out the expression status of Oligodendrocyte Lineage transcription factor 2 (OLIG2) and its role in cGBM, and help researchers get more details of cGBM molecular profiles and explore deeply in its biological mechanisms, and further, help practitioners develop precise therapy strategy for cGBM patients.

## Materials and methods

### Subjects

The database was reviewed at Huashan Hospital. The records of patients With Histopathological diagnosis of GBM (WHO IV) from 2005 to 2018 were included. Patients’ data was selected under the following criteria: (1) Age > 18y, (2) Without brainstem invasion, (3) Without any pre-operative treatment, (4) Surgical resection and pathological confirmation of GBM according to WHO 2016 criteria. The exclusion criteria were as following: (1) No histological diagnosis; (2) Pre-operative steroid usage.

All patients received craniotomy, and the tumor volume and the Extent Of Resection (EOR) was accessed with enhanced MR within 72 h after surgery. Post-operation adjuvant therapy was Radiotherapy including concurrent and sequential Temozolomide (TMZ) chemotherapy. The standard radiotherapy was administrated after surgery and performed toward the resection bed and residual lesions, (one fraction daily, 5 days per week).^13^ The dose of radiotherapy was 59.2–64.8 Gy in 30 fractions. The dose of concurrent TMZ was 75 mg/m^2^/day and sequential TMZ was 150–200 mg/m^2^/day 1–5 every 28 days for six cycles.^14^

All patients were continuously followed up by two doctors that were not involved in the therapeutic process. Clinical examinations and enhanced MR were performed at 3-month intervals or when tumor progression was clinically suspected. The overall follow-up duration ranged from Jan 2005 to Mar 2020 in our study. This retrospective study was approved by the Medical Ethics Committee of Huashan Hospital, Fudan University (KY2015-256), and was carried out in accordance with the Declaration of Helsinki.

### Tissue collection, IHC staining and evaluation

The tissue was collected during surgery. The samples were rinsed with phosphate buffer saline, fixed with formalin, and embedded in paraffin. The paraffin-embedded samples were further made into 3 μm GBM tissue slices to perform an immunohistochemistry assay using methods previously described.^15^ Primary monoclonal antibodies against the following antigens were used: Olig2 (Genetex, Texas, US).

Stained tumor samples were analyzed according to an Immunohistochemistry (IHC) score system. The IHC score was graded in a 5-stage intensity scale (0: ≤10%, 1: 11–20%; 2: 21–30%; 3: 31–60% and 4: > 60%).^16^ The average IHC scores were obtained based on ten randomly selected fields in each slice. Scores were defined as follows: at 0‒2 were low expression, at 3‒4 were high expression. Immunohistochemical features were evaluated by two independent doctors blindly, and then were confirmed by a pathologist.

### Statistical analysis

Patient and tumor characteristics were summarized using descriptive and percentage methods. Continuous data were described using the mean and standard deviations for parametric values. Categorical variables were described in frequencies and were compared using the Chi-Square test. Kaplan-Meier method was used to draw survival curves and a log-rank test was used for group comparison. Multivariate Cox proportional hazard models were used to investigate the contribution and Hazard Ratios (HR) of each prognostic factor on OS of cGBM patients. All analysis was performed by SPSS 20.0 (IBM Corporation, Armonk, New York. USA). For all statistical tests, *p* < 0.05 were considered statistical significance.

## Results

### Clinical characteristics of cGBM

73 cGBM patients were included in this study, the summary of the patients were shown in Table 1. Patients’ age was ranged from 18 to 72 years (mean 49.95 ± 14.66 years), including 49 males and 24 females. Tumor diameter varied from 1.4 to 4.6 cm (mean volume: 14.99 ± 1.55 cm^3^). As the cGBM located in posterior fossa and caused a ‘mass effect’, these patients experienced characteristic symptoms including vertigo and nausea (33/73, 45.2%), gait disturbances (25/73, 34.2%) and nystagmus (11/73, 15.1%). OLIG2 protein mainly accumulated in the cell nuclei. The staining scored high (3‒4) in 42 patients (42/73, 57.5%). 63 patients received gross total resection; 41 patients received adjuvant therapy.

**Table 1:** Demographic characteristics and of cGBM patients.

	cGBM (*n* = 73)
Age at diagnosis (%)	
≤ 40	42 (57.5%)
> 40	31 (42.5%)
Gender (%)	
Male	49 (67.1%)
Female	24 (32.9%)
Tumor diameter	
≤ 3	43 (58.9%)
> 3	30(41.1%)
EOR (%)	
Subtotal resection	10 (13.7%)
Gross tumor resection	63 (86.3%)
OLIG2 expression	
High	42 (57.5%)
Low	31 (42.5%)
Adjuvant therapy	
Yes	41 (56.2%)
No	32 (43.8%)

### OLIG2 expression and clinicopathological features in cGBM

**Fig. 1:**
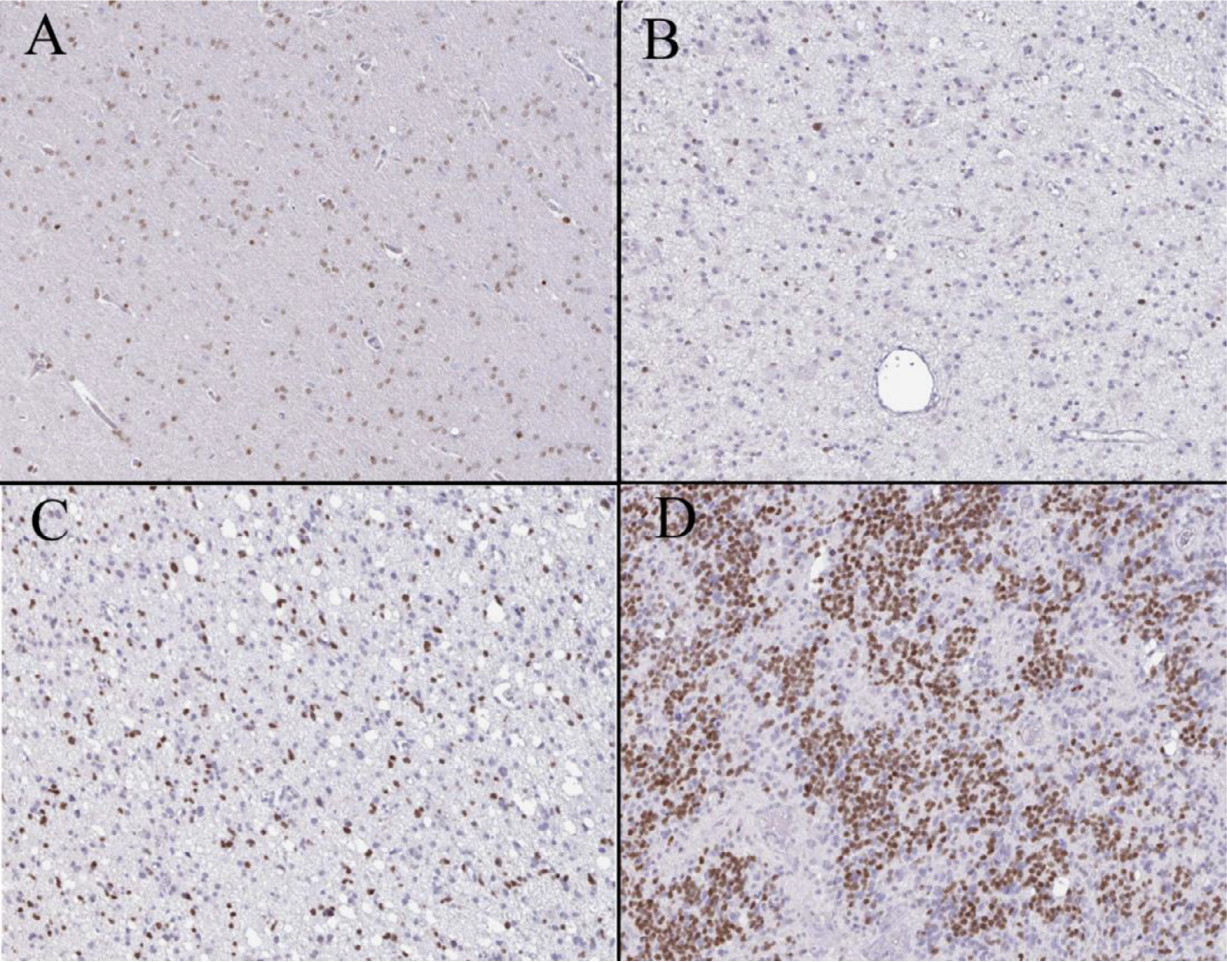
OLIG2 expression on immunohistochemistry. The intensity of OLIG2 is varied from 0 to 3 in Figure A to Figure D (magnification  × 400-fold).

The representative images of OLIG2 staining is shown in Fig. 1. Statistical analysis revealed that patients with high OLIG2 expression had a better alive rate (alive ratio: 70.6% vs. 29.4%, *p* = 0.04). The detailed correlation of OLIG2 expression level to clinicopathological features of these cGBM patients was listed in Table 2.

**Table 2:** Correlation of OLIG2 expression level to cGBM patients’ demographic characteristics.

Characteristics	Cases (*n* = 73)	OLIG2 Expression		*p*-value
		High (*n* = 42)	Low (*n* = 31)	
Age at diagnosis (%)				0.64
≤ 40	42	23(54.8%)	19 (45.2%)	
> 40	31	19(61.3%)	12 (38.7%)	
Gender (%)				0.80
Male	49	29(59.2%)	20(40.8%)	
Female	24	13(54.2%)	11 (45.8%)	
Tumor diameter				0.63
≤ 3	43	26(60.5%)	17(39.5%)	
> 3	30	16(53.3%)	14(46.7%)	
EOR				1.00
Gross resection	63	36 (57.1%)	27(42.9%)	
Subtotal resection	10	6(60%)	4(40%)	
Recurrence				0.20
Yes	51	32(62.7%)	19(37.3%)	
No	22	10(45.5%)	12(54.5%)	
Outcome				0.04
Death	40	18(46.2%)	21(53.8%)	
Alive	33	24(70.6%)	10(29.4%)	

### Prognostic value of OLIG2 expression in cGBM patients

**Fig. 2:**
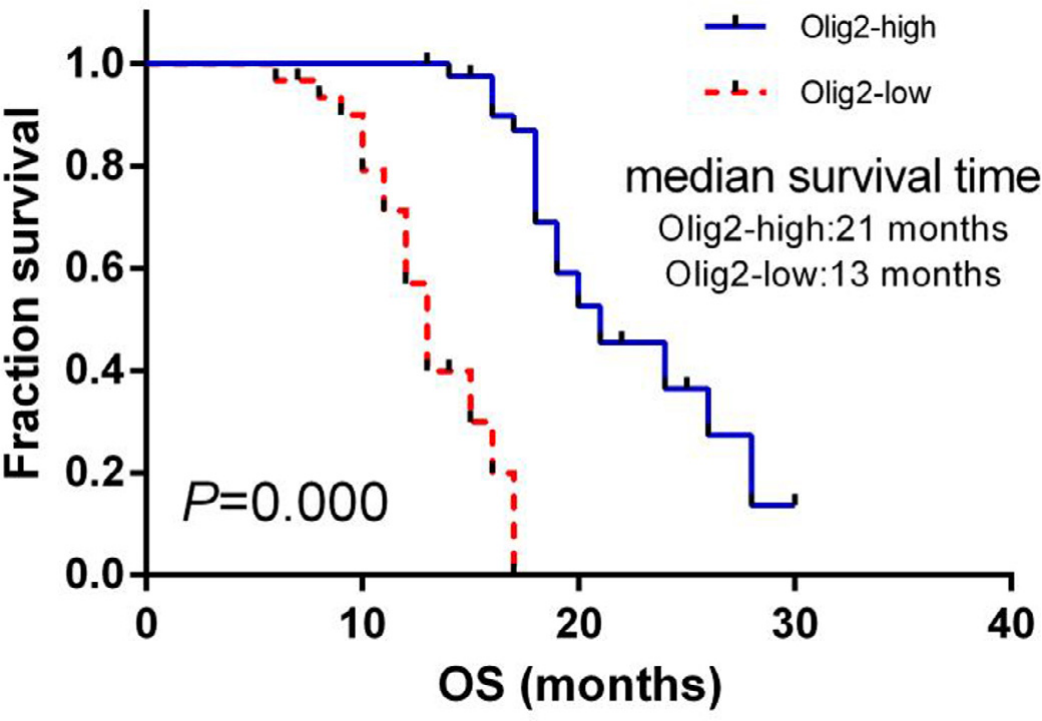
Kaplan-Meier analysis showed the high expression of OLIG2 had favorable OS for those cGBM patients.

Log-rank test was performed to access the factors that may interfere with patients’ OS, including age, gender, tumor diameter, resection extent, OLIG2 expression level, and adjuvant therapy in cGBM patients. Patients with high OLIG2 expression had favorable OS as compared to those with low OLIG2 expression (Log-rank = 48.65, *p* < 0.001, Table 3). The median survival time of patients with OLIG2 high expression was longer than those with low expression (21 vs. 13 months, *p* = 0.000) as indicated in Fig. 2.

**Table 3:** Kaplan-Meier analysis results of the OS of cGBM patients.

Prognostic parameters	Log-Rank	*p*-value
Age	0.06	0.81
Sex	0.07	0.79
Tumor diameter	1.50	0.22
EOR	29.64	0.00^a^
Olig2 expression	48.65	0.00^a^
Adjuvant therapy	49.05	0.00^a^

a *p* < 0.05.

Univariate Cox regression showed the subtotal resection (HR = 5.82, 95% CI 1.59‒21.26, *p* = 0.01), low OLIG2 expression (HR = 5.82, 95% CI 1.25‒27.14, *p* = 0.03), and without adjuvant therapy (HR = 5.16, 95% CI 1.29‒20.70, *p* = 0.02) significantly affected on the OS of cGBM patients (Table 4). Multivariate Cox regression also showed subtotal resection (HR = 3.89, 95% CI 1.23‒12.26, *p* = 0.02), low OLIG2 expression (HR = 5.26, 95% CI 1.13‒24.59, *p* = 0.04) and without radio-chemo therapy (HR = 4.95, 95% CI 1.22‒20.00, *p* = 0.03) were independent poor prognosis factors (Table 4).

**Table 4:** Cox regression analysis for the risk factors on the OS of cGBM patients.

	Univariate analysis		Multivatiate analysis	
	HR (95% CI)	*p*-value	HR (95% CI)	*p*-value
Age	1.89 (0.81‒4.44)	0.14		
Sex	1.09 (0.77‒1.53)	0.63		
Tumor diameter	1.18 (0.57‒2.45)	0.66		
EOR	5.82 (1.59‒21.26)	0.01^a^	3.89 (1.23‒12.26)	0.02^a^
Olig2 expression	5.82 (1.25‒27.14)	0.03^a^	5.26 (1.13‒24.59)	0.04^a^
Adjuvant therapy	5.16 (1.29‒20.70)	0.02^a^	4.95 (1.22‒20.00)	0.03^a^

a *p* < 0.05.

## Discussion

The cerebellar GBM is located in infratentorial space. The original location of GBM might determine the unique molecular mechanism of tumorigensis and clinical characteristics.^4^^,^^5^^,^^11^^,^^17^, ^18^, ^19^, ^20^ However, due to the limited sample size, no significant biomarkers were found in cGBM patients that correlate with their prognosis. In this present study, as the clinical cohort of 73 cGBM patients was the largest series in Asians to date, the results may give people some clues for further understanding of cGBM. From statistical analysis, the results indicated that a high OLIG2 expression level could be served as an independent factor for favorable prognosis for cGBM patients.

OLIG2 was reported high expressed in oligodendrogliomas and diffuse cerebellar gliomas.^21^^,^^22^ OLIG2 is important for maintaining the stem status of glioma and can activate cell proliferation machinery to promote tumorigenesis,^23^^,^^24^ and can also oppose the tumor suppressor p53 by direct transcriptional repression of p53-induced cell cycle inhibitor p21.^25^ The presented study revealed that OLIG2 was high expressed in 57.5% (42/73) cGBM patients, consistent with previous research,^21^^,^^26^ which seems associated with tumor malignancy.

As OLIG2 was recognized as a tumor promote factor in the research mentioned above, people may tend to take it as an indicator for poor prognosis for patients’ OS. Interestingly, statistical results in the presented study showed that cGBM patients with low OLIG2 expression had significantly shorter OS than those with high OLIG2 expression and had a higher risk of mortality (HR = 5.26). Considering other findings of previous research, the results of the present study may explainable. It has been revealed that proneural to mesenchymal transition is often associated with more aggressive GBMs.^27^^,^^28^ Researchers also found that OLIG2-knockdown glioma stem cells, although accompanied by tumor growth rate reduction, exhibit mesenchymal characteristics such as increased invasion and drug resistance.^23^^,^^29^ It also found that patients’ OLIG2 becomes low after adjuvant therapy, but has a significantly shorter time to recurrence and survival.^30^ These findings suggest that despite the OLIG2 expression promoting tumor growth, it suppresses the transformation of the glioma subtype from proneural to the worse mesenchymal type. This transformation results in the insensitiveness of glioma to adjuvant therapy.

The log-rank test indicated that gross total tumor resection and adjuvant therapy as well as high OLIG2 expression would contribute to the OS of cGBM patients in our present study. These results confirmed that adjuvant therapies are beneficial besides surgical resection of tumors as previous reports indicated.^31^ Meanwhile, OLIG2 expression level may be used as an indicator for tumor relapse and the effectiveness of adjuvant therapy. Cox regression analysis showed gross total tumor resection, high OLIG2 expression level and adjuvant therapy were independent favorable factors for the OS of cGBM patients. These results also remind us to further the research in tumor genome profiles and molecular characteristics besides surgical intervention for better understanding and treatment of this life-threatening disease.

As a single-center, retrospective study, there were also limitations. Not all patients received the standard adjuvant therapy plan, so OLIG2 expression levels on patients’ prognosis for those subgroups were not further analyzed because of the small sample size. In addition, OLIG2 expression levels after adjuvant therapy were not analyzed for this is a retrospective study.

## Conclusions

The study of 73 cGBM patients brings interesting results. Besides EOR and adjuvant therapy, OLIG2 expression level could be used as an independent prognostic factor for the OS of cGBM patients. Further investigation on the mechanics of OLIG2 in promoting cGBM would help researchers get a better understanding of this type of glioma. Meanwhile, a larger cohort of patient series and further studies are needed to get the whole profile of cGBM.

## CRediT authorship contribution statement

**Jia Zhou:** Funding acquisition, Conceptualization, Writing – original draft. **Ling-Fei Shi:** Data curation. **Zheng Wang:** Formal analysis, Data curation. **Min Li:** Formal analysis, Data curation. **Jin-Seng Zhang:** Writing – review & editing. **Ying Mao:** Conceptualization, Data curation. **Wei Hua:** Writing – review & editing.

## Declaration of Competing Interest

The authors declare that they have no known competing financial interests or personal relationships that could have appeared to influence the work reported in this paper.
